# Hospice and Palliative Care during Disasters: A Systematic Review

**DOI:** 10.3390/healthcare11172382

**Published:** 2023-08-24

**Authors:** Barbara Plagg, Julia Ballmann, Michael Ewers

**Affiliations:** 1 Charité—Universitätsmedizin Berlin, Corporate Member of Freie Universität Berlin and Humboldt-Universität zu Berlin, Institute of Health and Nursing Science, 13353 Berlin, Germanymichael.ewers@charite.de (M.E.); 2 Institute of General Practice and Public Health, College of Health Care Professions–Claudiana, Lorenz-Böhler-Straße 13, 39100 Bolzano, Italy

**Keywords:** hospice and palliative care, end-of-life care, public health emergency, disaster, disaster preparedness, crisis

## Abstract

Providing and maintaining hospice and palliative care during disasters poses significant challenges. To understand the impact of disasters on the provision of hospice and palliative care and the disaster preparedness initiatives in the field, a systematic review was undertaken. Eligibility criteria for the selection of studies were: peer-reviewed original research papers addressing HPC during disasters published between January 2001 and February 2023 in English. The databases CINAHL, MEDLINE, APA PsycInfo, APA PsycArticles, and SocINDEX were searched with textword and MeSh-terms between October 2022 and February 2023. The Mixed Methods Appraisal Tool (MMAT) was used to assess the quality of the studies. Content analysis was performed. The results are presented in the form of a narrative synthesis. Of 2581 studies identified, 57 met the inclusion criteria. Most studies were published recently on the impact of the COVID-19 pandemic. Four main themes were identified in the literature: disruption of the system, setting-specific differences, emotional challenges, and system adaptation. Overall, strategies to tackle hospice and palliative care needs have been poorly integrated in disaster preparedness planning. Our findings highlight the need to strengthen the resilience of hospice and palliative care providers to all types of disasters to maintain care standards.

## 1. Introduction

Disasters are massive disruptions to the functioning of a community or society due to hazardous events in conjunction with conditions and exposure that exceed their capacity [1,2,3] (Table 1). Globally, disasters are increasing in both complexity and magnitude due to a variety of mixed natural or human-made causes and increased vulnerabilities [4,5]. The Sendai Framework for Disaster Risk Reduction outlines four priorities for action to prevent and reduce disaster risks: (i) understanding disaster risk; (ii) strengthening disaster risk governance to manage disaster risk; (iii) invention in disaster reduction for resilience; and (iv) enhancing disaster preparedness [6]. With regard to health systems worldwide, the World Health Organization (WHO) recommends investing in a comprehensive all-hazards emergency preparedness framework taking into account the full scope of disasters [7], since the scale and severity of a disaster is strongly impacted by the socio-cultural and geopolitical setting of the affected population and by the type of threat [8].

In disaster contexts, with the surge of deaths and pressure on health services, the importance of hospice and palliative care (HPC) and the ethical obligation to provide comprehensive end-of-life care (EOLC) has been widely recognized, especially since the establishment of a WHO working group in 2016 to develop guidelines for HPC provision in humanitarian emergencies and crises of all types [8]. Although people affected by different types of emergencies in different settings require different kinds of care, according to the WHO, seven palliative care (PC) principles apply to any kind of emergency [8]. These PC principles are widely considered a key component of comprehensive HPC under adverse circumstances [3,8,9,10].

Healthcare systems are overall doubtlessly strained during disasters and providing or maintaining HPC services in the event of a disaster can be particularly challenging. However, a lack of empirical evidence with regard to HPC-specific barriers, challenges, and strategies in times of disasters has been reported, as well as little synthesis of existing evidence [11,12], likely due to limited prioritization and the primacy of lifesaving during disasters, data collection challenges, ethical considerations, and lack of resources and awareness. Systematic analyses of existing literature are essential to identify knowledge gaps and to anticipate the impacts of hazards in order to develop disaster-resilient models of HPC. Accordingly, the present review aims to provide a synthesis of the current literature, addressing the impact of disasters on the provision of HPC by identifying, among others, barriers, care needs, practices, and strategies.

## 2. Methods

The methodology adopted was a systematic literature review in accordance with the PRISMA-guidelines [13].

### 2.1. Eligibility Criteria

This systematic review included articles that focused on the impact of disasters on the provision of HPC and the disaster preparedness initiatives in the field. Further eligibility criteria for the selection of studies were: (i) peer-reviewed articles addressing HPC during disasters, (ii) original research papers, (iii) published between 2001 and February 2023, and (iv) in English. The start date was intentionally set to capture the post-9/11 period. All study designs (quantitative, qualitative, mixed- or multi-method) were eligible for inclusion. Studies were excluded if they (i) did not report the impact of disasters on HPC or (ii) were not original and peer-reviewed research papers (e.g., editorials, commentaries, opinions, policy papers).

### 2.2. Information Sources and Search Strategy

Within this systematic review, a comprehensive database search was performed including quantitative, qualitative, and mixed-method studies. The following databases were searched: (i) MEDLINE, (ii) CINAHL, (iii) APA PsycInfo, (iv) SocINDEX and (v) APA PsycArticles. The literature search was conducted using medical subject headings (MeSH 2022) and an additional keyword strategy. Due to the diffuse nature of this subject and its terminology, diverse MeSH terms and relevant text-words were used.

Although PC, hospice care (HC), and EOLC are often used with little distinction in the literature, they do have relevant conceptual differences: PC refers to specialized symptom-oriented comprehensive care for people with a no longer causally treatable illness to enhance a person’s quality of life throughout the course of the disease. Hospice care (HC) focuses on holistic comfort care and quality of life of a person with a terminal condition, including various social, spiritual, and everyday interventions. EOLC is directed toward the comprehensive care of people who are nearing the end of life, with great emphasis on the dignity of the person, their spirituality, and the maintenance of social relationships. As these concepts are all relevant to the care of patients with serious and/or terminal illnesses and those at the end of life, they were all included in the search (Appendix A). In the analysis of the papers, we use the terms that the authors themselves have used in their work. When we summarize results in which all or several approaches are included, we use the overarching term HPC.

The keywords were linked with the Boolean operator “and“ to increase precision and refine the search, limiting the results to records containing both terms (Appendix A). Additional studies were identified by free-text search and screening reference lists of included studies.

### 2.3. Selection Process

After duplicates were removed, titles and abstracts were reviewed independently by BP and JB against the inclusion criteria. To improve efficiency, we used Rayyan [14], a web-based systematic review automation software, which allows multiple “blinded” authors to collaborate. The full texts of potentially eligible articles were then retrieved for further review. Full texts were read and analyzed for eligibility by BP and JB and each record identified for inclusion was assessed for quality (see Section 2.5).

### 2.4. Data Extraction

Data were extracted from the primary source by two members of the research team (BP, JB) and organized in a formal synthesis matrix to facilitate a comprehensive analysis, including the following information: (i) authors, (ii) country where the study was conducted, (iii) study design, (iv) sample, and (v) findings/major themes. Initial pilot testing was performed on a subset of studies to ensure consistency between the two researchers (BP, JB) who met regularly to discuss challenges, refine the extraction process, and address discrepancies.

### 2.5. Quality Appraisal

Quality was assessed using the Mixed Methods Appraisal Tool 2018 (MMAT) since it provides, within a single tool, methodological quality criteria for different designs, including qualitative research, randomized controlled trials, non-randomized studies, quantitative descriptive studies, and mixed methods [15,16,17,18]. The MMAT includes two initial screening questions assessing the clarity of the research objective/question and the appropriateness of the method. Studies receiving a “no” are not feasible for further appraisal. The tool contains a subset of five questions for each category of study design (e.g., qualitative, quantitative randomized trials, quantitative non-randomized, quantitative descriptive, and mixed methods), the three response options include “yes” (criterion met), “no” (criterion not met), and “can’t tell”.

In contrast to an older version, where an overall score could be calculated by dividing the number of criteria met by four, the current version of the tool (2018) does not provide a score to be calculated, since it is discouraged to use metrics by the current literature in appraisal tools. Also, there is no cut-off value characterizing low- vs. high-quality studies since the categories are arbitrary. Thus, even when studies meet only three out of five categories, they may be included if the overall quality and the content-related added value are verifiable. BP and JB independently analyzed the quality for each article and any disagreements were resolved through discussions between the investigators.

### 2.6. Data Synthesis

The method of synthesis was based on a combination of textual narrative analysis [19] and content analysis. This hybrid method was chosen because it allows a more comprehensive understanding of the many eligible studies. BP and JB started by conducting a textual narrative analysis to gain a preliminary description of the results of the included studies. As patterns across studies began to emerge, the researchers moved on to thematic synthesis by systematically categorizing and organizing extracted data from the selected studies to identify themes and relationship among the findings.

## 3. Results

### 3.1. Study Selection

**Figure 1:**
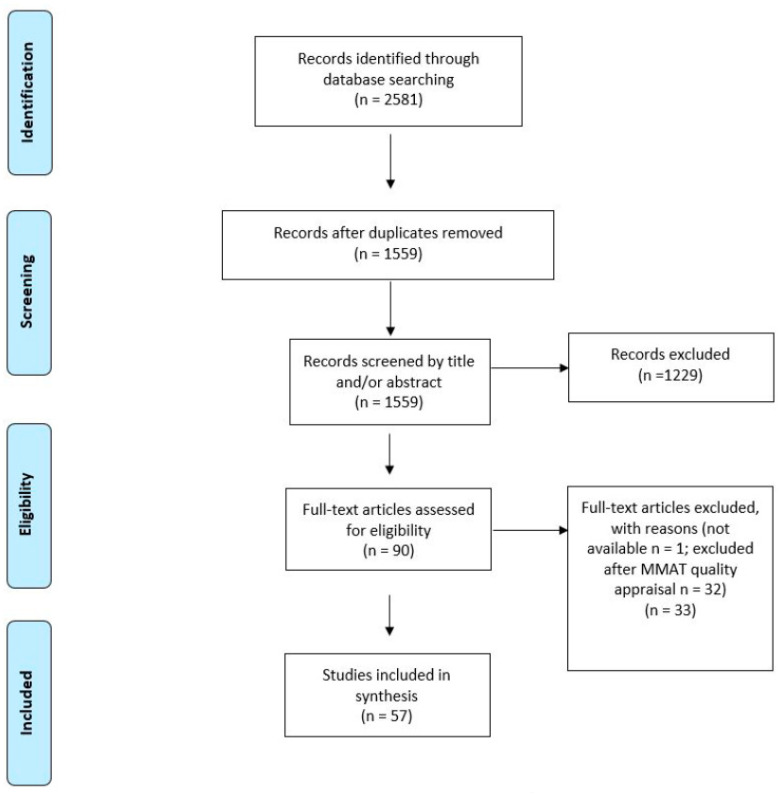
PRISMA Flowchart for the selection of studies.

A total of 57 papers met the inclusion criteria (Figure 1). Most of these papers were published since the outbreak of SARS-CoV-2 in 2020 (*n* = 51) and dealt with the situation of HPC and EOLC during the pandemic (Table 2). In the pre-pandemic era, there were only a few original research papers about HPC during disasters, with a focus on humanitarian crises and the SARS epidemic in 2003 [20,21,22,23,24]. These studies emphasized the importance of HPC during disasters. The authors agree on the need to include HPC in disaster management plans and to involve all healthcare professionals in the development of these plans. Despite these calls for preparation, our findings presented in the following subchapters indicate that HPC systems worldwide were largely unprepared when the COVID-19 pandemic broke out.

### 3.2. Study Characteristics

The following table presents an overview of the studies included in the analysis.

**Table 2:** Overview of the included studies (*n* = 57).

Author	Country & Year	Design	Sample
Aaronson et al. [25]	USA 2021	Interview study/ semi-structured interviews	ED and PC clinicians (*n* = 31)
Baker Rogers et al. [26]	USA 2022	Online survey	Members of the AAHPM and the HPNA (*n* = 83)
Becqué et al. [27]	Netherlands 2022	Interview study/in-depth narrative interviews	Purposively sampled bereaved relatives of patients who died during the COVID-19 Pandemic (*n* = 25)
Beltran-Aroca et al. [28]	Spain 2021	Retrospective observational cohort study	PC internal management database including all cancer patients treated in the period 2018–2021 (*n* = 1967)
Bradshaw et al. [29]	United Kingdom 2022	Qualitative multiple case study using semi-structured interviews	PC professionals (*n* = 24)
Bradshaw et al. [30]	United Kingdom 2021	Online survey	Palliative/hospice services (*n* = 277)
Chen et al. [20]	Taiwan 2006	Descriptive study	Comparison of in-patient admissions during 2002 and 2003 from the National Health Insurance Research Database with before-and-after comparisons
Chisbert-Alapont et al. [31]	Spain 2021	Cross-sectional descriptive study	Nursing professionals (*n* = 238)
Chou et al. [32]	Taiwan 2020	Cohort study	All patients cared for at Taipei Hospital from 2019 to 2020 (*n* = 19,900)
Costantini et al. [33]	Italy 2020	Cross-sectional telephone survey	Purposively sampled hospices (*n* = 16)
Doherty et al. [24]	Bangladesh 2020	Cross-sectional study with closed-ended interview questioning	Convenience sampling of individuals with serious health problems (*n* = 156) and caregivers (*n* = 155)
Dunleavy et al. [34]	United Kingdom 2021	Online multinational cross-sectional survey	Hospice and specialist PC providers (*n* = 458)
Finuf et al. [35]	USA 2022	Online survey	Members of palliative medical teams among 14 hospitals (*n* = 64)
Fish and Lloyd [36]	Scotland 2022	Interview study/in-depth narrative interviews	PC doctors (*n* = 8)
Franchini et al. [37]	Italy 2022	Survey/semi-structured telephone interviews	Home palliative care professionals; physicians (*n* = 15) and nurses (*n* = 15)
Garcia et al. [38]	Brazil 2022	Descriptive, cross-sectional survey with an online questionnaire	Healthcare professionals (*n* = 336)
Garner et al. [39]	United Kingdom 2022	Mixed-method	Online survey with UK hospices (*n* = 143); qualitative interviews with hospice professionals (*n* = 24)
Gerlach et al. [40]	Germany 2022	Interview study/ semi-structured interviews	Patients (*n* = 15) and family caregivers (*n* = 16)
Gonella et al. [41]	Italy 2022	Descriptive interview-study/inductive thematic analysis	Healthcare professionals (*n* = 21)
Hanna et al. [42]	United Kingdom 2021	Interview study/ semi-structured interviews	Health and social care professionals supporting dying patients (*n* = 16)
Hanna et al. [43]	United Kingdom 2021	Interview study/ semi-structured interviews	Bereaved family members (*n* = 19)
Hanna et al. [44]	United Kingdom 2022	Interview study/semi-structured interviews	Family carers (*n* = 26) and care home staff (*n* = 16)
Hasson et al. [45]	United Kingdom 2022	Online census survey	Managers of hospices (*n* = 150)
Haydar et al. [46]	USA 2020	Retrospective single-center study	Patients with COVID-19 (*n* = 242)
Hunt et al. [21]	Canada 2020	Interview study/ in-depth interviews	Humanitarian policy-makers and healthcare professionals (*n* = 24)
Jansky et al. [47]	Germany 2021	Interview study/focus groups/guided interviews	Representatives of SPHC teams (*n* = 18) and stakeholders (*n* = 5)
Kates et al. [48]	USA 2020	Cross-sectional survey	Hospice and PC workforce (*n* = 36)
Klinger et al. [49]	Germany 2022	Interview study/semi-structured expert interviews	People involved in pandemic management as a staff member attached to a healthcare facility or public institution (*n* = 41)
Lalani et al. [50]	USA 2022	Interview study/ qualitative interviews	PC professionals in rural areas (*n* = 15)
Leong et al. [22]	Singapore 2004	Interview study/semi-structured interviews	PC professionals (*n* = 8)
Lin et al. [51]	Taiwan 2021	Online cross-sectional survey	Subscribers (*n* = 1551) and organizational members (*n* = 185) of APHPCN
Macchi et al. [52]	Italy 2021	Qualitative study	Patients (*n* = 108) and carers (*n* = 90)
Mitchell et al. [53]	United Kingdom 2022	Cross-sectional online survey	Community nurses (*n* = 387); general practitioners (*n* = 156) and ‚other’ (*n* = 16)
Mitchinson et al. [54]	United Kingdom 2021	Semi-structured telephone interviews	Healthcare workers (*n* = 22/100 interviews mentioning death)
Nestor et al. [55]	Ireland 2021	Cross-sectional study using standardized questionnaires	Healthcare professionals (*n* = 250)
Nyblom et al. [56]	Sweden 2022	Interview study/semi-structured interviews	Patients (*n* = 22) and carers (*n* = 17)
Obata et al. [57]	USA 2020	Descriptive study	Clinical characteristics and comparisons between patients (*n* = 225) with COVID 19 with and without PC team consults
Oluyase et al. [58]	Multinational 2021	Online survey	Palliative care services (*n* = 458; UK *n* = 277; rest of Europe *n* = 85; rest of world *n* = 95)
Onwuteaka-Philipsen et al. [59]	Netherlands 2021	Observational online questionnaire survey	Healthcare professionals (*n* = 747)
Pastrana et al. [60]	Multinational 2021	Semi-structured online survey	PC professionals (*n* = 77) from 41 countries
Prokopová et al. [61]	Czech Republic 2022	Cross-sectional study	Healthcare professionals working in intensive care units (*n* = 313)
Rowe et al. [62]	USA 2021	Interview study/semi-structured interviews	PC clinicians (*n* = 25)
Sabolish et al. [63]	USA 2022	Retrospective exploratory study	Patients with PC consult (*n* = 174) and patients without PC consult (*n* = 152)
Samala et al. [64]	USA 2021	Cross-sectional study	Healthcare professionals (*n* = 64)
Schallenburger et al. [65]	Germany 2022	Interview study/ semi-structured online focus groups	Healthcare workers (*n* = 31)
Schloesser et al. [66]	Germany 2021	Post-bereavement online survey with free text options	Bereaved relatives (*n* = 81) from people who died during the pandemic with and without SARS-CoV2
Schneider et al. [23]	Switzerland 2018	Interview study/in-depth interviews	Expatriate health workers (*n* = 15) working with Médecins sans Frontières
Schoenherr et al. [67]	USA 2020	Evaluation of a pilot program for proactive identification of PC needs	Patients with COVID-19 (*n* = 29)
Seibel et al. [68]	Germany 2022	Interview study/qualitative interviews	Manager and health care workers (*n* = 29)
Selman et al. [69]	United Kingdom 2022	Open-web survey	Bereaved people (*n* = 711)
Sleeman et al. [70]	United Kingdom 2022	Cross-sectional online survey	PC services (*n* = 277)
Strang et al. [71]	Sweden 2020	Descriptive national registry data study	All registered patients who died of COVID-19 either in nursing homes or hospitals
Tanzi et al. [72]	Italy 2020	Single holistic case study design	Physicians (*n* = 9) and nurses (*n* = 22)
Varani et al. [73]	Italy 2021	Online survey	PC physicians and nurses (*n* = 145) working in home assistance compared to data collected in 2016 in the same setting (*n* = 179)
Wind et al. [74]	Denmark 2022	Semi-structured telephone interviews	Family carers (*n* = 15)
Yildiz et al. [75]	Netherlands 2022	Open observational online survey	Bereaved family members and friends (*n* = 393); relatives who lost a family member or friend at home (*n* = 68); in a hospital (*n* = 114), nursing home (*n* = 176) or hospice (*n* = 35)
Zheng et al. [76]	China 2020	Cross-sectional study	Healthcare professionals (*n* = 281)

### 3.3. Results of Syntheses

During the pandemic, single-center studies prevailed, while multi-center and mixed method surveys with larger sample sizes were rarer (Table 2). Most of the included articles were from Europe (*n* = 35), followed by the USA (*n* = 11), Asia (*n* = 6), Canada (*n* = 1), and South America (*n* = 1), and two multinational surveys were included. The content analysis identified four main themes (Table 3): (i) disruption of the system, (ii) setting-specific differences, (iii) emotional challenges, and (iv) system adaptation.

#### 3.3.1. Disruption of the System

##### Underutilization and Shift from Hospitals to Home Services

While there has been an increase in deaths during crises, a seemingly paradoxically underutilization of HPC services in hospitals has been reported [20,21,42,46]. During the pandemic, this was mainly due to a shift towards services providing care where requests increased [34,37,45,70]. The reasons for this shift appear manifold: Foremost, PC patients themselves did not want to be admitted because they were afraid of catching the infection. Furthermore, they wanted to stay close to the people who were important to them [56,58]. An overall reduction in referrals to hospitals from primary care was observed, as they appear to have been carried out only in particularly complex clinical cases [28,58]. Additionally, reallocation of resources toward lifesaving patient care and the perception of peoples’ EOLC and HPC needs as secondary to other challenges further led to HPC underapplication [49].

Compared to other pathologies, COVID-19 patients had lower utilization of PC. The possibility of having an EOLC discussion and dying with someone present was negatively affected by hospitalization [46,71]. Where HPC teams were involved, their outreach into the hospital and the community was reported to be helpful regarding patient care and family support. Furthermore, the HPC teams could ease the health workers’ psychological distress by helping to clarify advance directives and minimize unwanted resuscitative efforts [25,34,57,61,62,63,64,72]. Altogether, the underutilization of PC services during the pandemic was negatively perceived by experts and contrasted with the WHO’s “seven principles”.

##### Disruption of Social Connectedness

As different as disasters are, so are the associated challenges. However, during a public health emergency due to an infectious disease outbreak, the loss of social connectedness is a key theme, as described back in 2003 during the SARS-CoV outbreak and repeated on a large scale during the COVID-19 pandemic [22,45,60]. The implementation of strict infection prevention and control measures (IPC) lead to a disruption of social connectedness at multiple levels: between patients, families, healthcare workers, and communities [22]. As a result of restricted visiting to hospitals and nursing homes, relatives relied on connecting virtually with their family, which could, however, only happen when facilitated by creative and equipped health and social care professionals—which was not always the case [43]. However, the use of digital tools in health care increased rapidly during the COVID-19 pandemic. Despite this development, a discrepancy was described between the level of information desired by the relatives and what was provided in practice. This discrepancy may have been due to a lack of time and communication skills of the professionals in the field [43]. In general, both health professionals and family caregivers preferred in-person communication over technology-based communication because of its many shortcomings (e.g., frequent misunderstandings, “missing pieces”, lack of body language, etc.). ICT-based communication was at best perceived as a complementary option [61].

##### Dehumanization of Care

While measures in reaction to the COVID-19 pandemic varied amongst countries (see Section 3.3.4), strict visitor restrictions were negatively associated with healthcare staff’s appreciation of EOLC and the dying process [59]. Where family members were unable to plan for death, say farewell, or engage in traditional bereavement and death rituals, the risk for complicated and extended grief, anxiety, and depression, as well as for aggressive behaviors, distrust, and uncertainty increased [27,41]. Overall, IPC measures diluted health worker’s ability to provide care in accordance with their core values, resulting in experiences of moral distress, internal conflicts, and potential burnout [29,36,61]. Indeed, a major task for HPC providers during the pandemic was to continually manage the IPC-associated constraints on an organizational and individual level.

##### Lack of Resources

Several studies have reported that HPC services, in particular, lacked staff during disasters. On top of the already scarce professionals due to pre-existing staff shortages, absence due to illness and quarantine became a problem and volunteer support declined sharply. From the responding services within the CovPall study [58] who had volunteers, 79% used them much less during the pandemic. Material shortages included lack of medications, PPE, and other equipment, with charity-managed PC services being especially badly equipped [33,51,58]. A lack of opioids was reported by Médecins sans Frontières during various humanitarian emergencies [23,24]. In the CovPall study, however, a shortage of anaesthetic drugs was noted during the pandemic [58]. Overall, due to resource constraints, HPC services during the crisis had to rely on “quick fixes” and improvisation to provide fast low-cost solutions for their patients [34].

##### Lack of Information and Expertise

The lack of disaster-related protocols and setting-specific guidelines for HPC services in different settings, already described before the pandemic [22], became a major challenge during the COVID-19 outbreak [33,35]. It was notable that the pandemic response lacked consideration of HPC issues, including pre-determined responsibilities and trained specialist expertise in disaster mitigation strategies, as well as clarity in communication structures between macro, meso, and micro levels [49]. Without formal systems or guidelines to identify patients in need, HPC is often underused, especially in in-patient settings [25].

The definition of HPC expertise during the pandemic, especially in quickly formed COVID-19 PC units, was sometimes rather broad, as not all staff were trained PC experts [31,49,76]. Overall, with PC being only scarcely considered in disaster response, it was left to individuals with or without PC expertise at the micro level to balance ICP measures and care needs, while coordination from macro levels was lacking or only slowly developed [49].

#### 3.3.2. Setting-Specific Differences

HPC operates within numerous different contexts. In some studies, the specific setting was not explicitly indicated (e.g., studies that involved bereaved family members, irrespective of whether the patients passed away at home, in a hospital, or within a hospice facility, and surveys involving HPC providers working across different settings).

##### Preferred Place of Death

In general, hospitals and care homes are the least favoured place to die, especially during the pandemic [40,69]. Overall, dying at a place other than home, with medical and psychosocial care and visits being restricted, was associated with a lower likelihood to evaluate the place of death as appropriate by family members and healthcare staff, and was associated with challenges in the bereavement process [50,59,69,71].

##### Home Palliative Care during Disasters: Between Benefit and Burden

Due to an overall shift away from hospital care to home and community care, there was an increased demand for HPC outside of institutionalized care settings. As a response to this demand, home HPC flexibly reconfigured services, redeployed staff, and introduced new policies to minimize virus transmission [45]. Overall, post-bereavement analysis indicated that relatives of patients who died at home felt less burdened by the pandemic situation than relatives of patients who died in institutions, such as nursing homes or hospitals [66].

##### Cultural, Organizational, and Funding Differences in Disaster Response

Disaster reactions and mitigation strategies vary based on socio-political dimensions, healthcare system structure, and the extent of the disaster. Pandemic strategies thus ranged from countries with repeated lockdowns, loss of services, and social interactions, leading to negative emotional responses in the HPC sector [52], to countries with minor restrictions, where HPC workers found the impact of the disaster noticeable, but bearable [56]. Also, some health systems were widely unprepared to implement telehealth interventions, while others reported positive experiences [47]. Even crisis-experienced Asian countries lacked preparation, as the HPC sector was not equipped with advanced disaster management plans [20]. As well as country-specific differences, organizational and funding differences had an impact on disaster management: charitably managed HPC appeared especially fragile as their services were less integrated with national health services [39] and had a greater likelihood of material shortages [58].

#### 3.3.3. Emotional Challenges

Emotional challenges were a common theme in the available post-pandemic literature and many studies reported a wide spectrum of emotions while balancing HPC between disaster pressure and core values of care [42]. Overall, emotional challenges were framed by the availability of human, material, and knowledge resources, the patient’s vulnerability, visitation policies, and self-efficacy. There were concerns about contracting the virus and passing it on to vulnerable family members, challenges in supporting patients isolated from their relatives, the need to adapt to ever-changing protocols, difficulties in communication associated with the wearing of PPE, and reduced direct peer support [37,43,55,60,65]. Redeployed professionals felt less equipped and confident, even after “fast-tracked training” about PC [42].

Many studies reported that the practical and emotional challenges caused distress by destabilizing professional identity, which, in turn, may have impacted on the provision of patient care. However, the literature also highlighted that HPC workers adjusted their goals, developed staff resilience, showed commitment to their role, and were empowered by engaging in new forms of care and human connection [50,54]. Some studies reported high work satisfaction, professional self-confidence, and feeling supported by the (initial) national outpouring of goodwill towards front-line workers [36,37,55,73]. Interestingly, an Italian study reported that the frequency of burnout decreased during the peak of the pandemic, while psychological distress was significantly worse [73]. Another study found a possible explanation for this phenomenon: PC workers were observed to use avoidance techniques to reduce burnout perceptions; however, these maladaptive coping behaviors could have future negative effects, such as increased depression or post-traumatic symptoms [35].

For family caregivers, the pandemic situation led to emotional challenges as they struggled between worrying that a COVID-19 infection would lead to the patients’ untimely death, and living the limited time left to the fullest possible [40,56,74]. Relational aspects were of utmost importance for the relatives, and, where social connectedness was disrupted, care was considered “inhuman” [27]. Only a few studies examined the patient’s perspective: They expressed fears of infection risk, wasting precious time, insufficient healthcare resources, and abnormal death, while the patients’ already existing HPC needs remained unchanged during the pandemic [40,56].

#### 3.3.4. System Adaptation

Health systems were largely unprepared for the COVID-19 outbreak and plans and protocols were mostly lacking. However, HPC services in hospitals, communities and home care settings adapted dynamically to the various new challenges and restrictions; they tried to balance IPC measures and HPC needs, and adopted low-cost solutions [30,33,34,58,60]. In response to staff shortages, the improvisational and adaptive efforts of the system included new forms of collaboration, such as ad hoc establishment of COVID-19 PC units, increased involvement of specialized PC consult services for educating clinical staff, symptom management communication, and care guidance [51,58,68]. Additionally, staff worked longer hours, took greater responsibility, day-care services were closed, hospice beds were reduced, new staff were rapidly trained, and nonclinical staff were used [53,58]. Primary healthcare providers, which had previously experienced tensions with specialist PC teams, had the opportunity during the pandemic to strengthen mutual relationships through interprofessional training and collaborative practice approaches [53]. In response to shortages of equipment, PC services contacted various stakeholders, made their own supplies, and deployed staff to gather PPE as their main task, as they explored ways to use less material [58]. On a systemic level, services explored new strategies to proactively identify and meet the PC needs of all patients with COVID-19 at the intensive care unit or in the emergency department [25,67]. Against the loss of social connectedness, PC staff made efforts to re-humanize interactions and communication with patients and family by personal acts of care [36,54].

## 4. Discussion

HPC service provision, which is already resource-limited under conventional capacity, is inevitably further strained and under-resourced in crises and disasters [8,12]. During the COVID-19 pandemic, multiple dynamics were observed, such as a shift from the inpatient setting to home and community care services, a general lack of resources and/or its reallocation, a dehumanization of care, and the neglect of HPC services, both for those who were directly affected by the disasters (e.g., patients with COVID-19) and those who had pre-existing HPC needs. The vacuum of anticipating protocols led to “quick” fixes and improvisational strategies at the micro level of both inpatient and home care settings neglecting the holistic pretensions of standard HPC in adverse circumstances [8]. While the available literature highlights the dedication of healthcare professionals in providing the best possible service, our findings reveal that ad hoc disaster management strategies were often carried out by professionals who were lacking HPC expertise as well as lacking disaster education and training, and, often, the delivery of HPC could not be carried out in accordance with the very definition of HCP. In sum, under disaster pressure, HPC services worldwide adapted to a significantly lower level of care. Patient’s EOL and palliative needs were frequently treated as secondary to other challenges, resulting in these important resources being underutilized during disasters. The improvisational and altered ways of delivering HPC (if at all) led to multiple challenges amongst professionals, family caregivers, and patients since they often contrasted fundamental beliefs and values of HPC care.

The numerous studies included in the present review reveal that HPC has been given too little consideration in disaster management plans and/or providers were lacking education and training in disaster preparedness and/or palliative education. The Sendai Framework’s priorities, which specify that people with HPC needs should be included in disaster management policies and plans, remain mostly unmet [6]. However, with the pandemic, the utility and urgency of incorporating HPC into disaster preparedness plans has become evident and, thus, many, if not almost all, the included papers propose manifold strategies for strengthening crisis resilience and effective disaster planning. Indeed, our review clearly indicates that more efforts are needed in order to meet the goals set by the Sendai Framework for Disaster Risk Reduction 2015–2030 [6,77].

While hospitals may have disaster plans that encompass all wards, including PC units, they usually do not differentiate in terms of different patient groups or specialized care needs. Indeed, these plans seem to lack the specificity required for HPC approaches, while hospices and community-based services often lack any form of disaster preparedness. This is even more of a problem, as there was a shift from hospital-based services to primary care and community-based services during the pandemic. Indeed, the observed shift prompts a crucial reflection on healthcare organization and indicates that investing in home- and community-based HPC not only enhances the quality of EOL, HC and PC, but also contributes to the overall resilience and adaptability of healthcare systems, particularly, but not only, in times of crises. Strengthening community-based care may thereby help to alleviate the strain on hospitals and healthcare systems during disasters and may also enable a more distributed and flexible HPC delivery during disasters. Hence, the call for an intensive debate about healthcare organization, and extending this debate beyond hospitals to encompass, strengthen, and support hospices and home- and community-based settings, can play a pivotal role in fostering a comprehensive approach to disaster preparedness and building resilient HPC systems.

To develop crisis-resilient healthcare systems, hospice and palliative support needs must be included in systematic all-hazards disaster preparation efforts including a variety of disasters [10,11]. Planning should not only take micro levels into account, but should also consider, meso and macro levels and the respective organizational and federal structures. Since ethically, makeshift solutions appear mostly unsatisfactory and burdensome for all parties involved, potential conflicts of values (e.g., life-saving efforts vs. social connectedness) should be anticipated and addressed in concepts and guidelines. Staff training is an essential element in ensuring that an HPC team can provide care to patients and their families, particularly in the event of a disaster, as the team must be able to respond effectively and efficiently to ensure that patient care is not compromised. Regular training can help maintain their emotional resilience and ability to cope with the stress in challenging circumstances [78]. Additionally, strengthening outpatient and community- and home-based care services is necessary to tackle EOLC and HPC care needs under disaster pressure. Providing HPC services with adequate supplies and resources, and investing in tele-health, psychosocial, and practical support for patients, professionals and informal caregivers, is central to guarantee the continuity of HPC during disasters. However, not only in delivering direct patient care, but also in addressing public health needs, HPC providers are central in disaster mitigation, for instance, by providing training to other sectors of the healthcare system, by educating patients and family caregivers, by supporting community bereavement needs, and by providing public health surveillance [79].

The explosive increase in publications due to COVID-19 has had both positive and negative effects. On the one hand, it has generated new concepts and expanded our knowledge. On the other hand, hyper-prolific productivity and quick study set-ups may have resulted in research suffering from low validity and overall quality as evidenced by the exclusion of many papers. Nevertheless, even after sorting out weaker papers, robust evidence remains and existing models, policies, and strategies are now more than ever supported by evidence-based data. However, due to the very specific nature of COVID-19, the new body of literature may only be partially applicable to an all-hazards approach, making future all-hazards analysis necessary.

## 5. Limitations

This systematic review has several limitations: Since the identified empirical work relates to infectious health threads, with over 90% of all included papers being COVID-related, the results cannot be interpreted in terms of the all-hazards approach. It, therefore, remains largely unclear to what extent strategies exist to maintain HPC in the event of non-infectious disasters, such as heat waves, floods, or warfare, for example. Additionally, the included studies rarely assessed the patient’s perspective. Another limitation of the available literature is that authors framed the concepts of HC, PC, and EOLC differently and did not always provide a clear definition of their approaches, contributing to a lack of clarity. Moreover, papers were mostly from high-income countries and very few papers from low- and middle-income countries (LMICs) were included [8]. Since PC needs vary depending on sociopolitical and geopolitical backgrounds, and values regarding dying and death are culturally situated, the present evidence may not apply to health systems in LMICs. Additionally, due to reasons of space, we excluded literature focusing on telehealth solutions, non-original research (e.g., policy papers, letters, opinions), as well as the grey literature, and papers in languages other than English.

## 6. Conclusions

HPC occurs across various settings (e.g., hospitals, hospices, emergency care, nursing homes, and community care settings) and is an essential component of disaster response. However, while multiple barriers and challenges inevitably hamper the delivery of HPC during disasters, strategies to tackle EOLC and HPC needs have been poorly integrated in disaster preparedness planning. Strengthening the HPC sector is not a trivial matter, but a key disaster management strategy. With its special position in the medical care continuum, a well-prepared and equipped HPC system is able to tackle important public healthcare needs during disasters by supporting both healthcare systems and communities. Since, thus far, the resources and strengths of HPC are under-recognized and under-utilized, as, especially under crisis pressure, the primacy of lifesaving makes HPC needs less visible, they urgently need to be incorporated into disaster preparedness planning. Further emergency preparedness efforts, including all-hazards approaches and best practice analyses, and continued evaluation of HPC disaster planning, is needed to understand whether and how the transferral of the newly generated knowledge due to COVID-19 can be implemented into practical planning.

## Figures and Tables

**Table 1:** Classification of disasters.

Disasters	Group	Type (Examples)
Disasters triggered by natural hazards *	Biological	Pandemic, epidemic, insect infestation, animal stampede
	Geophysical	Earthquake, volcano, mass movement
	Meteorological	Storm
	Hydrological	Flood, mass movement
	Climatological	Extreme temperature, drought, wildfire
Man-made disasters	Warfare	National conflicts, international conflicts
	Socio-technical disaster	Technological disasters (e.g., leakage, toxic release, structure collapse), transportations disasters, production failure, public places failures

* The term “natural disaster” is misleading as it ignores the role humans play in this type of environmental disaster.

**Table 3:** Main themes and sub-themes related to disaster preparedness in HPC.

Main Themes	Sub-Themes
Disruption of the system	Underutilization and shift from hospitals to home services Underutilization of inpatient HPC services; COVID-19 patients had a lower utilization rate of HPC compared to other pathologies; Overall reduction in referrals from primary care, but increase in referrals of highly complex clinical cases; Increased utilization of outpatient HPC services providing hands-on care at home and in the community.
	Disruption of social connectedness IPC measures lead to disruption of connectedness at multiple levels; Increased use of information and communication technology (ICT).
	Dehumanization of care HPC workers torn between IPC measures and HPC core values; Increased risk for negative emotional reactions among professionals (e.g., moral distress) and informal carers (e.g., prolonged bereavement process).
	Lack of resources Lack of human resources; Material shortages (e.g., pain medications, PPE).
	Lack of information and expertise Lack of disaster-related protocols; Lack of HPC expertise.
Setting-specific differences	Hospitals and nursing homes Hospital and nursing homes appeared as the least favorable places to die during disasters.
	Home and community care Community HPC services flexibly reconfigured services due to increased demand.
	Cultural, organizational, and funding differences in disaster response Different reaction rates and mitigation strategies between countries and healthcare systems; Fragility of charitable hospice funding; No evidence from low- and middle-income countries (LMICs).
Emotional challenges	Increased emotional demands under disaster pressure for Professionals (e.g., increased workload, dehumanization of care, fear of infection, etc.); Redeployed professionals (e.g., uncertainty); Family carers (e.g., fear of loss of connectedness); Patients (e.g., fear of dying alone, fear of inappropriate medical care).
System adaption	Implementation of low-cost and ad hoc solutions especially at micro and meso levels Quick fixes and improvisation in response to staff shortages, lack of equipment, and lack of protocols; Ad hoc establishment of PC care units, interprofessional training, education and support of non-PC-staff; Adaptive efforts to re-humanize interactions.
System preparedness	Lack of PC-specific disaster management Despite calls for disaster preparedness, HPC appears up to date unprepared.

## Data Availability

The data presented in this study are available on request from the corresponding author.

## Appendix A

*MeSH-search-combinations (each combined with ‘AND’)*	
Palliative care; Palliative medicine; Terminal care; End of life care; Hospices; Hospice care; Hospice and palliative care nursing	Disasters; Eruption, volcanic; Earthquakes; Floods; Hurricane; Drought; Droughts; Typhoon; Avalanche; Cyclonic storms; Pandemics; Epidemics; Warfare and armed conflicts; Terrorism; Weapons; Radioactive hazard release
*Additional textword search in databases and alert* *(APA PsycArticles, APA PsycInfo, CINAHL, MEDLINE, SocINDEX)*	
(palliative care or palliative medicine or end of life care or terminal care or terminal care or hospice care or hospices or hospice and palliative care nursing) AND (disaster or crisis) AND (eruption* or earthquake* or flood* or hurricane* or drought* or cyclonic storm* or typhoon* or avalanche* or cyclone* or pandemic* or epidemic* or war* or armed conflict* or terrorism or weapon* or accident* or power failure or famine or civil war or war)	
